# Supplementation of Blackcurrant Anthocyanins Increased Cyclic Glycine-Proline in the Cerebrospinal Fluid of Parkinson Patients: Potential Treatment to Improve Insulin-Like Growth Factor-1 Function

**DOI:** 10.3390/nu10060714

**Published:** 2018-06-02

**Authors:** Dawei Fan, Yassar Alamri, Karen Liu, Michael MacAskill, Paul Harris, Margaret Brimble, John Dalrymple-Alford, Tim Prickett, Oliver Menzies, Andrew Laurenson, Tim Anderson, Jian Guan

**Affiliations:** 1 Department of Pharmacology and Clinical Pharmacology, School of Medical Sciences, Faculty of Medical and Health Sciences, University of Auckland, Auckland 1142, New Zealand; d.fan@auckland.ac.nz (D.F.); karen.liu@auckland.ac.nz (K.L.); 2 Centre for Brain Research, Faculty of Medicine and Health Science, University of Auckland, Auckland 1142, New Zealand; Tim.Anderson@cdhb.health.nz; 3 Brain Research New Zealand, A Centre of Research Excellence, New Zealand; m.brimble@auckland.ac.nz (M.B.); john.dalrymple-alford@canterbury.ac.nz (J.D.-A.); 4 New Zealand Brain Research Institute, Christchurch 8011, New Zealand; yassar.alamri@nzbri.org (Y.A.); michael.macaskill@nzbri.org (M.M.); 5 Canterbury District Health Board, Christchurch 8041, New Zealand; andrew.laurenson@westcoastdhb.health.nz; 6 Department of Medicine, University of Otago, Dunedin 9016, New Zealand; tim.prickett@nzbi.org; 7 Department of Medicinal Chemistry, School of Chemistry, University of Auckland, Auckland 1142, New Zealand; paul.harris@auckland.ac.nz; 8 Department of Psychology, University of Canterbury, Christchurch 8041, New Zealand; 9 Department of Geriatric Medicine, Auckland District Health Board, Auckland, 1142, New Zealand; OliverM@adhb.govt.nz; 10 Department of Neurology, Christchurch Public Hospital, Christchurch 8140, New Zealand

**Keywords:** cyclic Glycine-Proline (cGP), bioavailability of insulin-like growth factor-1 (IGF-1), Parkinson disease, blackcurrant anthocyanins, autocrine regulation, cerebrospinal fluid, central uptake

## Abstract

Background: Insulin-like growth factor-1 (IGF-1) function is impaired in Parkinson disease. Cyclic glycine-proline (cGP), a metabolite of IGF-1, is neuroprotective through improving IGF-1 function. Parkinson disease patients score lower on Hospital-associated Anxiety and Depression Scale after supplementing blackcurrant anthocyanins (BCA), which may be associated with IGF-1 function. We evaluated the changes of cGP and IGF-1 before and after the supplementation. Methods: Plasma and cerebrospinal fluid (CSF) were collected from 11 male patients before and after 28 day supplementation of BCA. The concentrations of IGF-1, IGF binding protein (IGFBP)-3, and cGP were measured using ELISA and HPLC-MS assays. The presence of cGP in the BCA was evaluated. Results: cGP presented in the BCA. BCA supplementation increased the concentration of cGP (*p* < 0.01), but not IGF-1 and IGFBP-3 in the CSF. CSF concentration of cGP was correlated with plasma concentration of cGP (R = 0.68, *p* = 0.01) and cGP/IGF-1 molar ratio (R = 0.66, *p* = 0.01). The CSF/plasma ratio was high in cGP and low in IGF-1 and IGFBP-3. Conclusion: cGP is a natural nutrient to the BCA. The increased CSF cGP in Parkinson disease patients may result from the central uptake of plasma cGP. Given neurotrophic function, oral availability, and effective central uptake of cGP, the BCA has the potential to be developed to treat neurological conditions with IGF-1 deficiency.

## 1. Introduction

Parkinson disease (PD) is the second most common neurodegenerative condition. Insulin-like growth factor-1 (IGF-1), a neurotrophic factor, plays an essential role in neuronal survival and brain functions. IGF-1 resistance, characterized by the increase of circulating IGF-1 with impaired IGF-1 function, plays a role in disease progression, cognitive impairment, and pathology of idiopathic PD [1,2,3,4]. Thus, changes of plasma IGF-1 have been under clinical investigation for monitoring IGF-1 function, in order to predict the prognosis and treatment responses in PD [5]. However, the measurable IGF-1 in plasma is largely inactive. The majority of plasma IGF-1 is bound to IGF binding protein (IGFBP)s, in which more than 75% is IGFBP-3 [6]. Binding to IGFBP-3 prevents IGF-1 from activating IGF-1 receptors and from being metabolized [6]. Thus, IGFBP-3 regulates IGF-1 function in both stimulatory and inhibitory manners, namely, autocrine regulation of IGF-1 [6]. Only a small amount of unbound IGF-1 (or free IGF-1) in plasma is bioactive, and the window of opportunity for directly detecting unbound IGF-1 is small, because free IGF-1 is either rapidly metabolized or internalized after interacting with IGF receptors. Therefore, plasma IGF-1 concentration does not represent the function of IGF-1. Nonetheless, the IGF-1 concentration in plasma is still frequently evaluated for indicating IGF-1 function [5,7]. As an alternative, the ratio of IGF-1/IGFBP-3 has been used for indicating ‘free’ bioactive IGF-1. Yet, the measurement includes a large amount of free IGFBP-3 [8]; thus, is still not a reliable representation of bioavailable IGF-1. 

A better representation of the bioavailability of IGF-1 is instead feasible by measuring the levels of cyclic glycine-proline (cGP), a metabolite from ‘free’, bioactive IGF-1 [8,9]. The cGP is formed from the N-terminal tripeptide of IGF-1 [7] after being cleaved [10,11,12] by an acid enzyme [13,14]. The N-terminal of IGF-1 is a primary binding site for IGFBP-3 [12], and cGP retains the same affinity for interacting with IGFBP-3 in a concentration-dependent manner [15]. The function of cGP is mediated through competition with the binding of IGF-1 to IGFBP-3 [15], in which the higher ratio of cGP/IGF-1 associates with a greater free IGF-1 and better IGF-1 function [15]. Administration of cGP protects the brain from ischemic injury in rats [15,16] by improving IGF-1 function [15]. A structure analogue of cGP also protects dopamine neurons from 6-hydroxydopamine-induced injury and improves long-term functional recovery in a rat model of PD [17,18]. 

High consumption of berry-fruits has been reported to be associated with a lower risk of PD [19]. In an open-label study [20], PD patients display a lower Hospital-associated Anxiety and Depression Scale (HADS) score after the supplementation with blackcurrant anthocyanin (BCA, 35% anthocyanins). However, the authors have disassociated the improved neuropsychological outcome with the supplementation; thus, the mechanism underlying the beneficial effect of BCA remains unclear. It has been reported that the effects of anthocyanin on preventing the apoptosis and cardiac dysfunction of diabetic rats are mediated by activating IGF-1 receptors and signaling pathways [21]. In addition, purple wheat is high in anthocyanins, and the effects of purple wheat on prolonging the life span of Caenorhabditis elegans are associated with the IGF-1 signaling pathway [22]. We, therefore, sought to examine the possibility of BCA intake on altering IGF-1 function by examining CSF and plasma levels of cGP, IGF-1 and IGFBP-3. 

## 2. Materials and Methods 

### 2.1. Recruitment and Clinical Information of Parkinson Patients 

**Figure 1:**
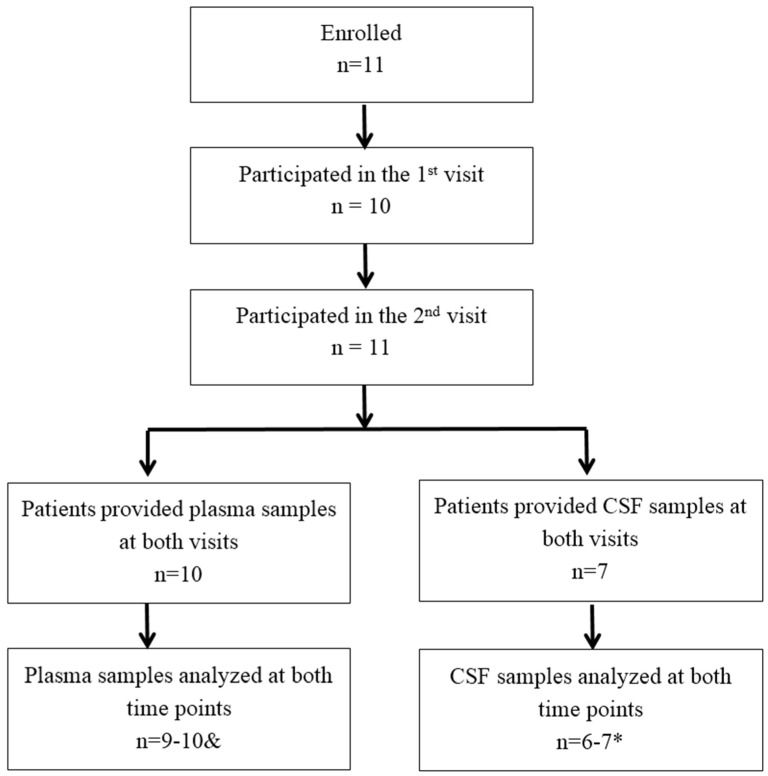
Flowchart of study population. *, ^&^ due to the amount of CSF samples available for analysis, seven pairs of CSF samples were analyzed for cGP and six pairs for IGF-1 and IGFBP-3. The data from one participant were excluded from statistical analysis due to a 15 times increase in CSF cGP and 19 times increase in plasma cGP after supplementation.

The recruitment and clinical background of the patients have been described in a previous publication [20]. Briefly, male PD patients were recruited from the Van der Veer Movement Disorders clinic and the patient database of New Zealand Brain Research Institute. Patients were eligible to enrol in the study irrespective of the stage of disease and time since diagnosis. The patients were aged 40 years or older and met the UK Brain Bank criteria for idiopathic PD confirmed by a movement disorders neurologist. The study was approved by the Upper South A Regional Ethics Committee (reference: URA/10/03/022). A flowchart shows the study population (Figure 1).

Patients were assessed using the Unified PD Rating Scale (UPDRS) parts III and also administered a battery of psycho-cognitive tests before obtaining the samples at first visit. These included the HADS, the Mini-Mental State Examination (MMSE), the Montreal Cognitive Assessment (MoCA), and the PD Questionnaire (PDQ-39). To avoid any learning effects, different versions of the tests were used for the second visit if available. Table 1 shows the clinical information and assessments of the patients prior to the trial. The majority of patients were diagnosed as idiopathic PD without obvious cognitive impairment. 

### 2.2. Trial Design

The current study analyzed the biological changes using the plasma and CSF that were collected from the clinical trial. The trial for clinical assessments has been partially reported [20]. Thus, we only included the unpublished information that was relevant to sample collections. The trial was run over two visits 28 days apart. During each visit, plasma and CSF samples were collected. Patients were instructed to consume a “low-anthocyanin diet” (i.e., white rice, white bread, tuna, chicken, coffee, and non-herbal tea) 12 h before each visit. Following the first visit, patients were supplemented with blackcurrant capsules over the next 28 days. The dose of BCA concentrate capsules (35% anthocyanins, Super Currantex^®^ 20, supplied by Vitality New Zealand, manufactured by Just The Berries Ltd., Christchurch, New Zealand) were 300 mg taken twice daily for four weeks. The last dose was taken during or just before the second visit. 

### 2.3. Samples Collection

#### 2.3.1. CSF and Plasma Samples

CSF samples were obtained by lumbar punctures. Around 8 mL of CSF was obtained during each visit. The CSF sample, collected in a plain tube, was transported on wet ice to Endolab, Christchurch, New Zealand, within 15 min of collection. The samples were then centrifuged at 3000 rpm for 15 min at room temperature, and the supernatant was aliquoted equally between two plain tubes and frozen at −80 °C within 30 min of sample-receipt. Blood samples were obtained via venipuncture of the antecubital fossa; 20 mL was divided equally between heparin and EDTA tubes. The samples were immediately transported on wet ice to Endolab within 45 min of collection. The samples were then centrifuged at 3000 rpm for 15 min at room temperature, and the plasma was aspirated into a plain tube and frozen at −80 °C within 30 min of sample-receipt.

#### 2.3.2. In Vitro Samples

To analyze the potential presence of cGP in the BCA, BCA was dissolved in Milli-Q water with 3 different concentrations of 5, 50, and 100 mg/mL. Each concentration has been analyzed in five duplicates. 

### 2.4. cGP Assays

The methodology has been previously described [8]. Briefly, cGP-d_6_ provided an internal standard for cGP assay. cGP-d_6_ (50 μL of 500 ng/mL) was added to 100 μL of plasma and vortex mixed. The solution was transferred to a 1 mL Phree phospholipid removal cartridge (Phenomenex, Auckland, New Zealand) contained in 4.5 mL tube; 500 μL of 1% formic acid in Acetonitrile was added to the cartridge and centrifuged at 1000 rpm for 5 min at 4 °C to enable the collection of the filtrate. The filtrate was dried using a vacuum concentrator (1.5 mTor for an hour, then 0.7 mTor for 45 min, at room temperature). The dried samples were reconstituted in 100 μL 10% methanol/water and transferred to an ultra-pressure liquid chromatography vial for quantitation, then centrifuged at 500 rpm for 5 min at 4 °C to sediment any remaining particulates. Standards prepared by spiking cGP into charcoal stripped human plasma, quality control samples, with cGP at two different concentrations, were utilized and then subjected to the same extraction procedure as the samples. 

### 2.5. High Performance Liquid Chromatography Mass Spectrometry Assay (HPLC-ms) 

The method has been described in our previous publication [8]. Briefly, the chromatography conditions consisted of a Synergy Hydro 2.5 µm column (Phenomenex) 100 × 2 mm with an initial mobile phase composition of 10% methanol/90% water flowing at 200 μL per minute with a column temperature of 35 °C. The mass spectrometry conditions consisted of electrospray ionization in positive mode with a voltage of 4000 V, a sheath gas flow of 30 psi, an auxiliary gas flow of 2 psi, and a capillary temperature of 250 °C. Fragmentation achieved with argon at 1.2 mTorr as the collision gas and a dissociation voltage of 35 V. The mass spectrometer ran in selective reaction monitoring mode with the following two transitions 155.1 → 70.2 m/z and 161 → 75.1 m/z utilized for cGP and cGP-d_6_, respectively. The retention time for both peaks was 3.6 min. Unknown samples were quantitated using the peak area ratio of cGP/cGP-d_6_ compared with the standard curve of known concentrations. 

### 2.6. ELISA

Plasma and CSF concentration of total IGF-1 and IGFBP-3 were measured using commercial ELISA kits (Crystal Chem, Chicago, IL, USA) according to manufacturer’s instructions. The assays were repeated four times in plasma samples and but only duplicated in CSF samples due to the limited amount of CSF available. 

### 2.7. Statistical Analysis

Paired t-test was used for analyzing the changes in cGP, IGF-1, IGFBP-3, and the ratio of cGP/IGF-1 before and after the BCA supplementation. Two tailed one samples t-test was used for analyzing the percentage changes of concentrations. One-way ANOVA was used for analyzing the concentration of cGP of the BCA. Correlations between the biological changes were calculated using Pearson tests. A *p*-value less than 0.05 is considered to be significant. Percentage change was calculated by the following formula: (value after supplementation − value before supplementation)/value before supplementation) × 100. The data were presented as mean ± SME and the percentage changes after supplementation. 

## 3. Results

### 3.1. Analysis of cGP Concentration in the BCA

**Figure 2:**
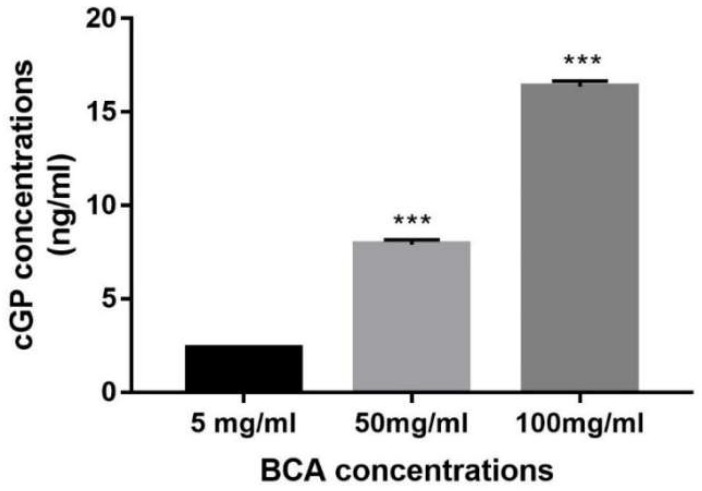
A dose-dependent increase of cGP concentration in the BCA. Data presented as mean ± SEM, *** *p* < 0.0001.

One-way ANOVA suggested the concentration of cGP was significantly different between the samples with different dose of BCA (*p* < 0.0001, n = 5, Figure 2), with a dose-dependent increase of cGP concentration in the BCA. Compared to the samples with low dose of BCA (5 mg/mL), the concentration of cGP was significantly increased in the samples with 50 mg/mL BCA (*p* < 0.0001, n = 5) and further increased when the BCA dose increased to 100 mg/mL (*p* < 0.0001, n = 5). 

### 3.2. CSF

**Figure 3:**
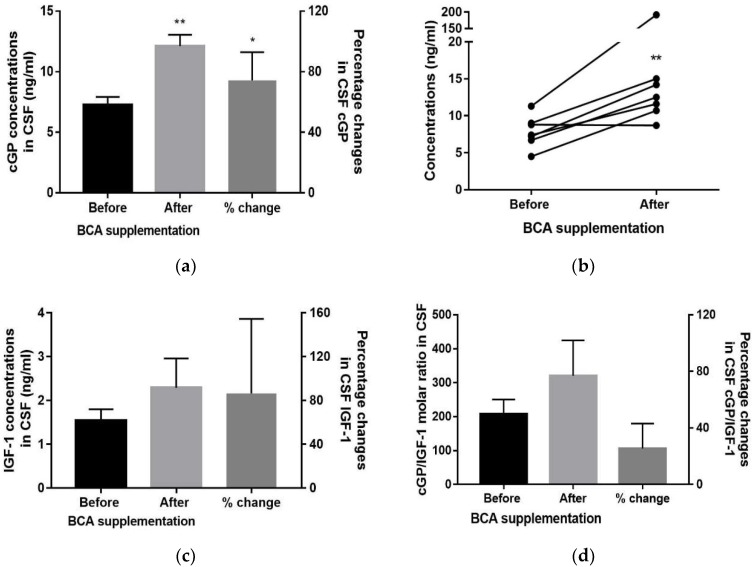
The changes in the CSF before and after supplementation of the BCA. (**a**) The concentration of cGP in the CSF was significantly increased after the supplementation compared to that prior to the supplementation. The percentage changes of cGP concentrations were significantly increased in the CSF. (**b**) There 6/7 paired samples showed an increase in cGP after supplementation, with one pair of samples increased by 16.9 times. cGP concentration of 1 pair of samples remained the same after the supplementation. (**c**) There was no statistical difference in IGF-1 concentration and percentage change of IGF-1 in the CSF. (**d**) There was no statistical difference in the cGP/IGF-1 ratio and percentage change of the ratio. Data presented as mean ± SEM and the percentage change from the baseline, * *p* < 0.05, ** *p* < 0.01.

There was a significant increase in the concentration of CSF cGP after BCA supplementation (from 7.27 ± 0.67 ng/mL to 12.12 ± 0.94 ng/mL, *p* < 0.01, n = 6, Figure 3a). The mean of percentage changes in cGP concentrations was increased by 74.36% after supplementation (*p* < 0.05, t(5) = 3.989). Amongst total seven pairs of samples, six of them showed an increase after supplementation (Figure 3b). One patient showed 16.9 times increase (11.30 ng/mL to 191.80 ng/mL) of cGP in the CSF, which has been eliminated from the statistical analysis as an outlier (15.7 times of mean, Figure 3b). One patient did not respond to the supplementation and the cGP concentration of this patient remained the same after the supplementation (from 8.8–8.7 ng/mL, Figure 3b). There was no significant change in the concentrations of IGF-1, cGP/IGF-1 ratio (Figure 3c,d), and IGFBP-3 (Table 2). 

### 3.3. Plasma

There were no changes in the concentrations of cGP, IGF-1, cGP/IGF-1 ratio (Figure S1), and IGFBP-3 following BCA supplementation (Table 2).

### 3.4. CSF vs. Plasma Concentration

Table 2 shows the relative values of cGP, IGF-1, and IGFBP-3 in CSF and plasma before and after supplementation. The CSF/plasma ratio was <1% in both IGF-1 and IGFBP-3 and 52% in cGP before supplementation, and increased to 71% after the supplementation (Table 2). 

### 3.5. Correlation Analysis

**Figure 4:**
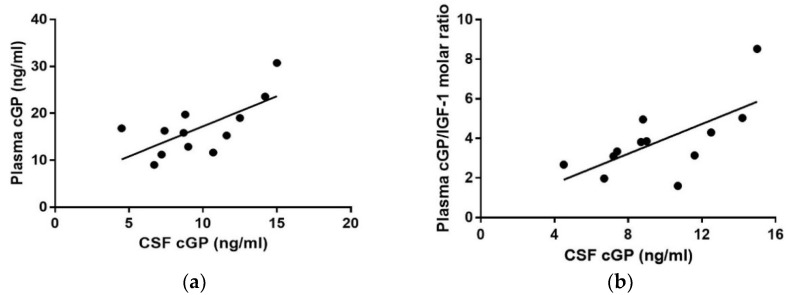
Correlation between the concentrations of cGP in CSF and plasma. (**a**) There was a significant positive correlation between CSF and plasma cGP (R = 0.68, *p* < 0.01). (**b**) There was a significant positive correlation between CSF cGP and plasma cGP/IGF-1 (R = 0.66, *p* < 0.01).

Pearson tests revealed strong correlation between the concentrations of cGP in CSF and plasma (R = 0.68, *p* = 0.01 Figure 4a, n = 12), as well as between the molar ratio of cGP/IGF-1 in plasma and cGP concentrations in CSF (R = 0.66, *p* = 0.01, Figure 4b, n = 12). 

There was no correlation in IGF-1 concentration between the CSF and plasma (R = −0.10, *p* = 0.75) and no correlation between cGP and IGF-1 concentration in both CSF (R = −0.13, *p* = 0.69) and plasma (R = 0.05, *p* = 0.85). 

## 4. Discussion

As a neuropeptide, cGP is a natural nutrient of the BCA. The supplementation of the BCA led to an increase of CSF cGP in PD patients, suggesting oral availability and effective brain uptake of cGP. The cGP concentrations were correlated between the CSF and plasma, suggesting the plasma cGP may be the potential source. Given the well-characterized function and mechanisms of cGP in brain protection, the supplementation of the BCA may be further developed for treating neurological conditions through a larger clinical trial. 

Neuroprotective effects of cGP have been demonstrated in a rat model with ischemic brain injury [16]. The treatment effects of cGP are mediated by improving IGF-1function by increasing bioavailable IGF-1 [15]. Administration of a structure cGP analogue, cyclic Glycine-2ally-Proline (NNZ 2591), after the onset of motor deficits, improves long-term motor function in a rat model of PD [18] and normalizes neuroplasticity in a rat model with acute memory impairment [23]. The effectiveness of cGP and cGP analogue in brain protection and functional recovery proves that cGP is a neurotrophic agent. Supplementation of the BCA, which has such neurotrophic nutrient, increased CSF cGP concentration in PD patients. The data provide the first clinical evidence for oral availability and effective central uptake of cGP following the intake of foods. 

cGP is a small and lipophilic molecule (192 d) and is able to cross the blood-brain barrier (BBB) in vivo [16]. Approximately 52% of plasma (endogenous) cGP found in the CSF before the supplementation, which increased to 71% after the supplementation. Given the ability of cGP crossing the BBB, the significant correlation between CSF and plasma concentration of cGP may suggest that the plasma cGP could be a likely source for the increase of CSF cGP, even though the supplementation did not change plasma concentration of cGP. We did not see a correlation between cGP and IGF-1 in the CSF, which could not exclude the possibility that a small part of CSF cGP forms from IGF-1 in the central nerve system (CNS), as the enzyme that cleaves IGF-1 also occurs in the CNS [14]. 

In contrast to cGP, the CSF concentration of IGF-1 was about 1% of that in the plasma, suggesting that CSF IGF-1 was largely independent of circulating IGF-1. IGF-1 is a larger molecule (7600 d) than cGP, with limited ability to cross the BBB [24]. Using post-mortem human brain tissue, we have recently reported that the BBB function is not compromised in most of the brain regions of idiopathic PD cases that show the absence of dementia-related pathology [25]. It is possible that the demand for trophic supports of degenerating brains promoted cGP transfer from plasma to CSF. Indeed, the direct administration of cGP to CSF protects brains from ischemic injury [15]. Thus, further increase of CSF cGP after supplementation of anthocyanin may potentially lead to the improvement of IGF-1 function in PD brains. Activating IGF-1 receptors and signalling pathways has been suggested to be the mechanism underlying the treatment effects of anthocyanin that prevents apoptosis and cardiac dysfunction in diabetic rats [21]. However, clinical benefits from BCA supplementation were not conclusive, as the changes of CSF cGP were not significantly correlated with the HADS scores (*p* = 0.18, R = 0.39, data not shown). Large clinical trials are essential to confirm the efficacy of BCA supplementation in clinical outcome of PD. 

The majority of plasma IGF-1 is inactive due to the binding to IGFBPs [6]. Nonetheless, the increase in plasma IGF-1 has been used for indicating IGF-1 resistances in PD patients [1]. However, such changes of IGF-1 in plasma are not always observed [26]. The increased plasma IGF-1 in PD may be an ineffective endocrine response to produce more circulating IGF-1. The limited central uptake of IGF-1 could be a contributing factor to IGF-1 resistance in PD patients [1]. Circulating IGF-1 may decline when the condition of PD deteriorates to the stage with the complication of cognitive impairment [5,27]. 

Apart from increasing IGF-1 production, namely, endocrine regulation, the IGF-1 function is also regulated by improving autocrine/paracrine regulations, particularly under the situation that IGF-1 production is insufficient [8,28]. The deficiency of IGF-1 could be the result of impaired autocrine regulation trough interacting with IGFBPs [8]. We have reported the role of cGP in regulating IGF-1 bioavailability by competing with IGF-1 binding to IGFBP-3 [15] under both physiological [9,29] and pathological conditions [8]. The competitive binding between IGF-1 and cGP is concentration-dependent, resulting in more cGP and more active IGF-1. Our observations from other clinical and experimental studies show that the increase of cGP and/or cGP/IGF-1 ratio is associated with weight changes in obese women [8], post-natal development [9], and spontaneous recovery in stroke patients [30]. Thus, the changes of plasma cGP and/or cGP/IGF-1 ratio may provide an additional indication of IGF-1 function. Autocrine regulation of IGF-1 may present in the CNS, which could be different from that in plasma [6]. Given limited access to CSF samples, a larger clinical trial is essential for determining whether plasma cGP and cGP/IGF-1 ratio would provide a reliable indication for IGF-1 function in PD and other neurological conditions with intact BBB. 

Even though the results were significant, this pilot study has a clear limitation due to small sample size. The interpretation of the results should be cautious until confirmation is received from large clinical trials. Total of 6 out of 7 patients had positively responded to the supplementation by showing increases of cGP in the CSF, suggesting the change is sensitive. One patient did not respond to the treatment and had the highest score of PDQ-39. With 10 patients in each group, we also detected a significant decrease of plasma cGP in hypertensive women and increase of cGP/IGF-1 ratio in obese women [8]. The longitudinal design used in current trial may eliminate some clinical variations and improve the sensitivity of the changes in plasma cGP and cGP/IGF-1 ratio [8]. The clinical research for evaluating cGP and cGP/IGF-1 ratio for the IGF-1 function is still in its infancy. If it confirmed through large trials, the changes of plasma cGP would help to individualize BCA intervention. 

## 5. Conclusions 

In conclusion, our study for the first time demonstrated that cGP, a neuropeptide, was a nature nutrient of the BCA and provided clinical evidence of oral availability and effective brain uptake of cGP after supplementation of the BCA. The increased cGP in the CSF of PD patients may be the result of central uptake of plasma cGP, so that IGF-1 function can be improved in PD brains. The changes of cGP and cGP/IGF-1 ratio in plasma might provide an additional indication for IGF-1 function in degenerative brains. 

## Figures and Tables

**Table 1:** Clinical information of 10 male PD patients before BCA supplementation.

Case	Age	Clinical Diagnosis	UPDRS III	MMSE	MoCA	HADS	PDQ-39
BM02BC	61	idiopathic PD	15	29	29	6	43.75
LE14BC	77	idiopathic PD	48	27	22	3	31.25
SE07BC	73	idiopathic PD	36	30	24	9	90.63
YY03BC	48	idiopathic PD	33	29	29	8	59.83
EK05BC	80	idiopathic PD	52	29	27	6	156.25
GD08BC	80	idiopathic PD	34	28	24	11	134.88
ED12BC	60	idiopathic PD	33	30	25	3	65.63
EY17BC	56	idiopathic PD	27	30	28	21	159.38
TN15BC	70	idiopathic PD	51	28	22	21	250
KT16BC	55	idiopathic PD	31	28	26	4	71.88

HADS, Hospital Anxiety and Depression Scale; MMSE, Mini-mental State Examination; MoCA, Montreal Cognitive Assessment; PDQ-39, PD Questionnaire; UPDRS-III, Unified Parkinson Disease Rating Scale-part three.

**Table 2:** CSF and plasma concentrations of cGP, IGF-1, and IGFBP-3 before and after BCA supplementation.

**Before the Supplementation**			
	CSF (ng/mL) Mean ± SEM (n = 6)	Plasma (ng/mL) Mean ± SEM (n = 9–10)	Ratio of CSF to plasma (%)
IGF-1	1.54 ± 0.26	179.04 ±14.89	0.86%
cGP	7.27 ± 0.67	13.96 ± 1.33	52.01%
IGFBP-3	26.16 ± 2.79	3038.92 ± 111.90	0.86%
**After the Supplementation**			
	CSF (ng/mL) Mean ± SEM (n = 6)	Plasma (ng/mL) Mean ± SEM (n = 9–10)	Ratio of CSF to plasma
IGF-1	2.29 ± 0.67	176.07 ± 14.13	1.30%
cGP	12.12 ± 0.94	16.92 ± 2.79	71.63%
IGFBP-3	27.69 ± 3.53	3029.09 ± 59.35	0.91%

## Supplementary Materials

The following are available online at http://www.mdpi.com/2072-6643/10/6/714/s1, Figure S1: The changes in the plasma following supplementation of BCA. There was no statistical change in cGP (a), IGF-1 (b) and the ratio of cGP/IGF-1 (c). Data presented as mean ± SEM and the percentage changes.
