# Supplementary material for: Supplementation of Blackcurrant Anthocyanins Increased Cyclic Glycine-Proline in the Cerebrospinal Fluid of Parkinson Patients: Potential Treatment to Improve Insulin-Like Growth Factor-1 Function
Source: Nutrients. 2018 Jun 2;10(6):714. doi: 10.3390/nu10060714 (PMC6024688; doi:10.3390/nu10060714)
Supplement: Supplementary file 1 [file nutrients-10-00714-s001.pdf]

Supplementary Figure 1:

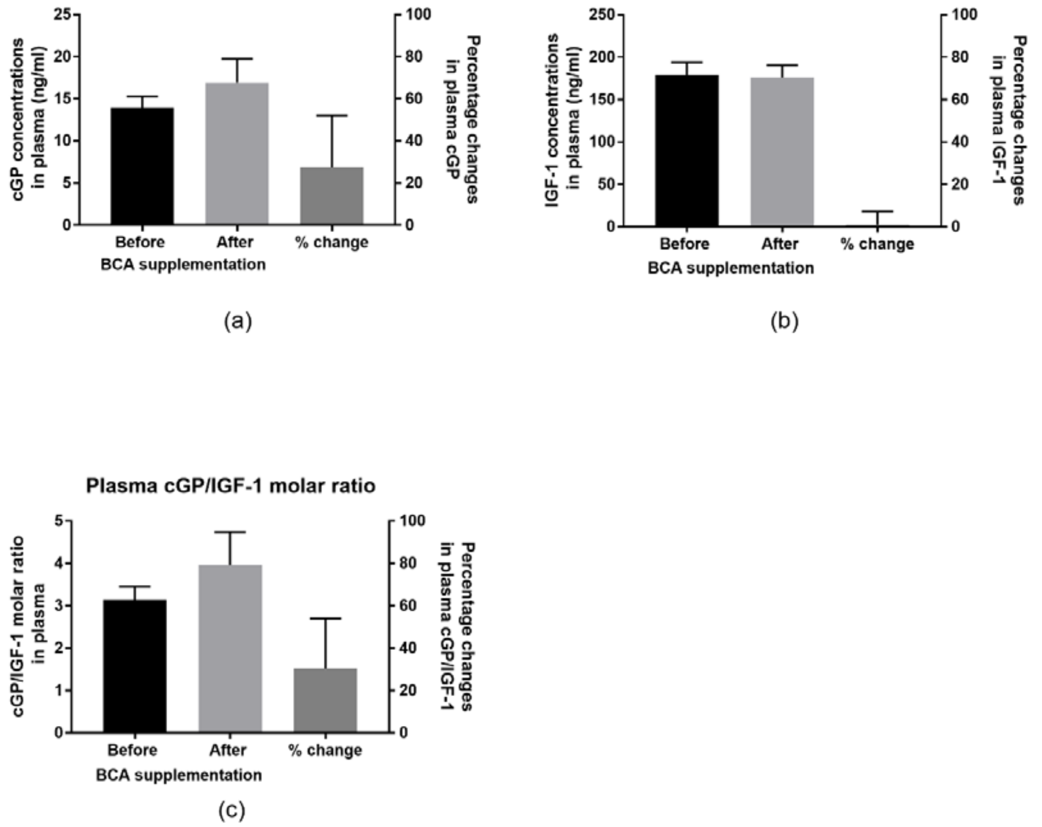

Supplementary Figure 1. The changes in the plasma following supplementation of BCA. There was no statistical change in cGP (a), IGF-1 (b) and the ratio of cGP/IGF-1 (c). Data presented as mean ± SEM and the percentage changes.
